# Microglia as potential key regulators in viral-induced neuroinflammation

**DOI:** 10.3389/fncel.2024.1426079

**Published:** 2024-07-11

**Authors:** Fatme Seval Ismail, Timo Jendrik Faustmann, Pedro M. Faustmann, Franco Corvace

**Affiliations:** ^1^Department of Neurology, Klinikum Vest, Academic Teaching Hospital of the Ruhr University Bochum, Recklinghausen, Germany; ^2^Department of Psychiatry and Psychotherapy, Medical Faculty, Heinrich Heine University, Düsseldorf, Germany; ^3^Department of Neuroanatomy and Molecular Brain Research, Medical Faculty, Ruhr University Bochum, Bochum, Germany

**Keywords:** microglia, virus, neuroinflammation, innate immunity, adaptive immunity

## 1 Introduction

### 1.1 Types of viruses involved in CNS infections

Viruses are the most frequently identified cause of central nervous system (CNS) infections (Said and Kang, 2023). A wide range of viruses including Herpesviridae, Enterovirus, Arbovirus, Rhabdovirus, Paramyxovirus, Retrovirus, Flaviviruses can lead to infections of the CNS such as meningitis, encephalitis or encephalomyelitis which occur either immediately or later after a couple of days, weeks or even years (Solomon et al., 2007; Bookstaver et al., 2017; Rocamonde et al., 2023).

### 1.2 Courses of viral-induced CNS infections

Different courses of viral-induced CNS infections are possible such as acute, long-term persistent (chronic or relapsing), asymptomatic and late-onset neuroinflammation.

### 1.3 Mechanisms of CNS infiltration by viruses

#### 1.3.1 Trojan horse strategy

Viral CNS infections manifest complex compared to infections in peripheral tissues, and thus viruses have developed multiple strategies to overcome the natural protective barriers of the CNS such as the blood-brain barrier (BBB) and the blood-cerebrospinal fluid (CSF) barrier. Multiple viral routes to reach the CNS are described (Rocamonde et al., 2023). Among the proposed mechanisms is a cell-associated transport using immune cells, so-called Trojan's horse strategy. Viruses like Human T-Leukemia Virus (HTLV)-1, Measles Virus (MeV), Natural Nipah virus (NiV), Epstein-Barr Virus (EBV) can infect peripheral dendritic cells, lymphocytes and macrophages which patrol the healthy CNS, indicating that also viral-infected-immune cells can infiltrate into the CNS and contribute to infection of CNS resident cells (Ousman and Kubes, 2012; Rocamonde et al., 2023).

#### 1.3.2 Direct infection of endothelial cells

Further mechanisms of CNS infiltration include a direct infection of endothelial cells by viruses or in an indirect way through endothelial cells by transcytosis, leading to BBB disruption with increased permeability to lymphocytes (Afonso et al., 2008).

#### 1.3.3 Nasopharyngeal route and axonal spread

Another route of CNS entry is through the nasopharyngeal route with infection of pulmonary epithelium e.g., in case of MeV with subsequent infection of lymphocytes and transmission to endothelial cells, or in case of NiV with direct infection of neurons in the olfactory bulb and using the anterograde viral transport to disseminate in the direction of the ventral cortex (Munster et al., 2012; Rocamonde et al., 2023). Furthermore, axonal spread of Herpesviridae has been demonstrated as an efficient way for CNS invasion. For example, Alpha-herpesvirus such as herpes simplex virus 1 (HSV1) can disseminate from infected neurons in both anterograde and retrograde directions, while viral material controls the spreading direction (Taylor and Enquist, 2015). Rabdoviridae including Rabies virus can also use anterograde and retrograde transport to gain access to the CNS, but there is no consensus on CNS entry and spreading routes for Flaviviridae such as West Nile Virus (WNV) (Taylor and Enquist, 2015).

### 1.4 Glial responses to viral-induced CNS infections

#### 1.4.1 Role of astrocytes and microglia

A recent review discussed the glial responses to viral-induced CNS infections (Rocamonde et al., 2023). Astrocytes, microglia and oligodendrocytes are counted among the glial cells as key players in CNS health and disease (Verkhratsky et al., 2023). Microglia and astrocytes contribute significantly to the innate immune system of the CNS, undergoing complex reactive remodeling dedicated to the defense of the CNS (Escartin et al., 2021; Paolicelli et al., 2022). Different forms of CNS pathology including neuroinfection result in astroglial reactivity/reactive astrogliosis which encompasses complex and variable structural, molecular and functional alterations of astrocytes (Verkhratsky et al., 2023). Microglia form the main group of resident immune cells in the CNS, which contribute to maintaining CNS health and homeostasis through core properties including surveillance, phagocytosis, and capacity for releasing soluble factors (Ransohoff and Perry, 2009; Kettenmann et al., 2011). Pathologic events result in reactive microgliosis and proliferation including microglial phenotypical changes from the homeostatic ramified into the reactive phenotype (Kettenmann et al., 2011; Paolicelli et al., 2022). Further, a connection between the microglial role during neuroinflammation and the development of neurodegenerative mechanisms was discussed, pointing toward a continuum between neuroinflammation and neurodegeneration (González-Scarano and Baltuch, 1999; Hickman et al., 2018). Direct infection, bystander activation, cell-cell interactions with involvement of glial cells belong to the mechanisms contributing to development of viral-induced neuroinflammation.

## 2 Microglia as potential key regulators in viral-induced neuroinflammation

### 2.1 Microglia as key regulators

#### 2.1.1 Microglial reactivity and immune response

**Figure 1:**
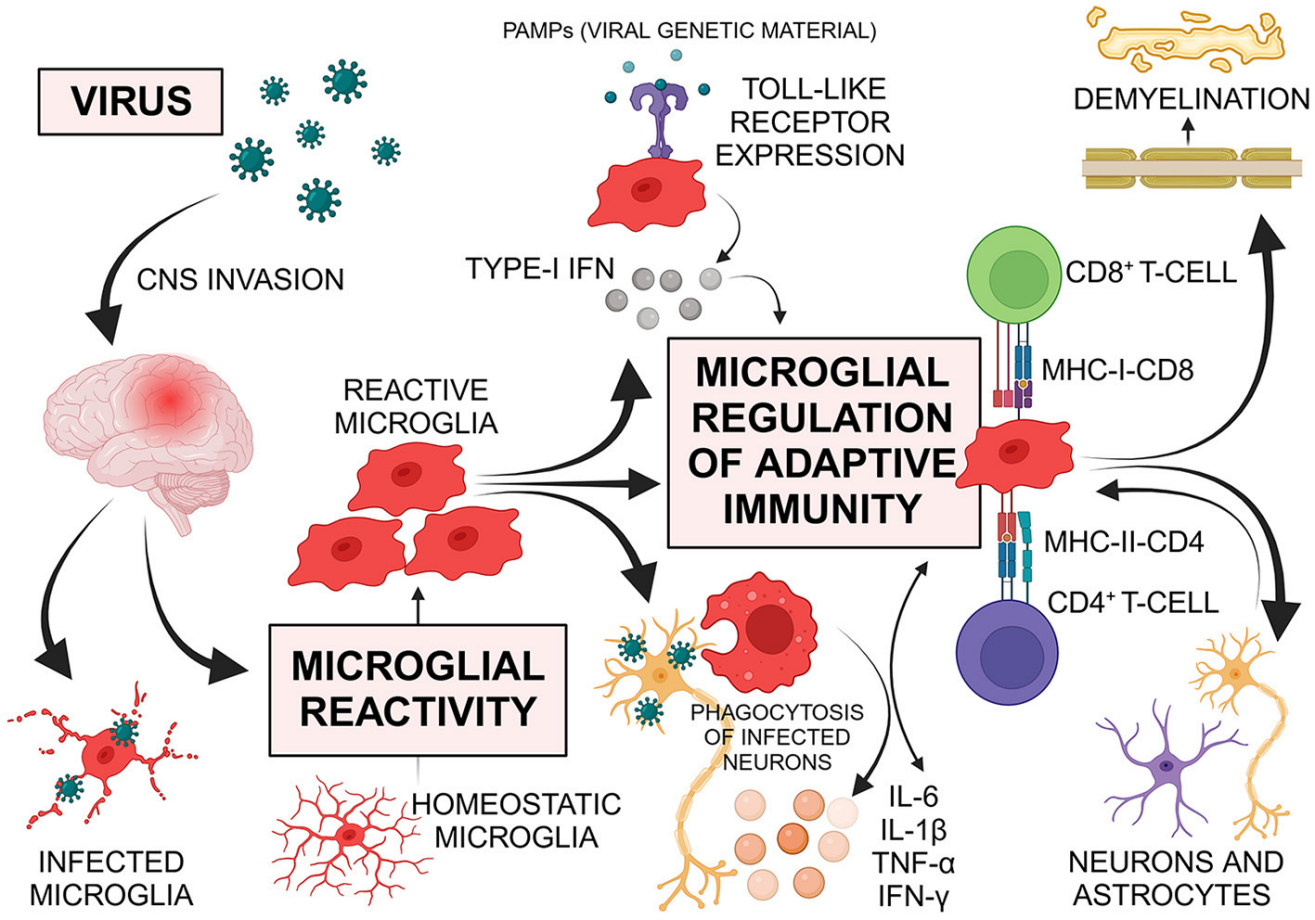
Viruses use multiple routes to invade the central nervous system (CNS). Upon entry, the first immune response is initiated by resident microglia. Microglia respond to viral infections through morphological reactivity, proliferation, phagocytosis of infected cells, antigen presentation, and various receptor expressions. They release chemokines and cytokines such as interleukin (IL)-6, tumor necrosis factor (TNF)-α, IL-1β, and interferon (IFN)-γ, which promote further innate and adaptive immune responses, including the regulation of T-cells. Microglia express various Toll-like receptors (TLRs) that recognize viruses and can trigger type-I IFN production, leading to the transcription of IFN-stimulated genes and regulating the antiviral immune response. As regulators of adaptive immunity in the CNS, microglia mediate T-cell responses. Antigen presentation by microglia is crucial for forming protective T-cell responses against infections or malignancies and for generating pathogenic, autoreactive T-cell responses in autoimmune conditions. The interaction between the T-cell receptor and major histocompatibility complex (MHC) molecules, which contain processed antigenic peptides, on the surface of antigen-presenting cells (APCs) is essential for this process. Reactive microglia, functioning as APCs, share features with peripheral macrophages and are vital components of both innate and adaptive immune responses. Upon reactivity, microglia up-regulate MHC and co-stimulatory molecules, with MHC class I stimulating CD8^+^ cytotoxic T-cells and MHC class II stimulating CD4^+^ T-helper cells. T-cell activation through antigen-presenting microglia can result in the production of IFN-γ and TNF, which further activate microglia to engulf healthy synapses post-infection. Despite the immune privilege of the CNS, viruses can induce autoimmune diseases through mechanisms such as bystander activation of autoreactive cells or molecular mimicry. Microglia contribute to demyelination, displaying both harmful and beneficial effects. Additionally, astrocytes and neurons regulate microglial function through various soluble factors, highlighting the importance of cellular interactions. PAMPs pathogen-associated molecular patterns; Created with BioRender.com.

In our opinion, we highlight the microglia as potential key regulators in viral-induced neuroinflammation (Figure 1). Microglia are involved in the control of non-specific inflammation/innate immunity as well as adaptive immune responses. In addition to their well-established phagocytic function, microglia contribute to innate immune functions, antigen presentation and CNS immunopathology (Aloisi, 2001; Borst et al., 2021). After the viruses enter the CNS, the first immune response occurs through the resident microglia. Depending on the type of microglial response, the course of viral-induced neuroinflammation could be defined e.g., direct infection with acute or sustained neuroinflammation, bystander activation of innate/adaptive immunity, viral clearance. Several neuropathological and experimental study findings demonstrated microglial reactivity and virus antigen detection in microglia during the different courses (acute/chronic or relapsing/asymptomatic and persistent/late-onset) of CNS infection, confirming that various viruses can infect microglia and/or lead to microglial immune response (Rocamonde et al., 2023). Otherwise, the early involvement of microglia in the viral-induced CNS-inflammation can determine the course and outcome.

### 2.2 Toll-like receptors in microglial reactivity

Accumulating evidence reveals that Toll-like receptors (TLR) are crucial in the regulation of innate immune responses to pathogens, recognizing a wide range of pathogen-associated molecular patterns (PAMP) such as bacterial and viral gene products (e.g., double-stranded RNA and DNA), bacterial peptidoglycan, lipopolysaccharide. Previous findings confirmed the expression of various TLRs by human adult microglia (Bsibsi et al., 2002). Viral gene products and glycoproteins recognized by TLRs in antigen-presenting cells can cause type-I interferon (IFN) secretion with subsequent transcription of IFN-regulated genes involved in antiviral immune response. Microglia TLRs recognizing viruses can also trigger type-I IFN production similar to dendritic/antigen-presenting cells, all this supporting the view that microglia are key regulators of immune responses in the CNS (Aloisi, 2001; Rocamonde et al., 2023).

### 2.3 Microglial response to viral infections

Along with morphological reactivity, proliferation, phagocytosis of infected cells and different receptor expressions, microglial response to viral infections of the CNS includes release of chemokines and cytokines such as interleukin (IL)-6, tumor necrosis factor (TNF)-α, IL-1β, IFN-γ, promoting further innate and adaptive immune responses (Furr and Marriott, 2012). The ratio between pro-inflammatory and anti-inflammatory mediators released by microglia can be crucial in demyelination e.g., TNF-α and IFN-γ secretion resulted in oligodendrocyte apoptosis and impaired proliferation of oligodendrocyte progenitor cells (Shi et al., 2015).

Microglia are regulators of T-cell mediated immune responses (Aloisi, 2001). Antigen presentation plays a crucial role in the formation of protective T-cell responses in case of infection or malignancy and of pathogenic, autoreactive T-cell responses in case of autoimmunity. For this purpose, the T-cell receptor and the major histocompatibility complex (MHC) molecules, containing processed antigenic peptides, on the surface of antigen-presenting cells interact with each other. Upon reactivity, microglia up-regulate both MHC and co-stimulatory molecules. MHC class I and MHC class II molecules lead to stimulation of CD8^+^ cytotoxic T-cells and CD4^+^ T-helper cells, respectively. During CNS infection, microglia act as antigen-presenting cells with consequent activation of T-cells producing IFN-γ and TNF, which in turn promotes microglia for engulfment of healthy synapses delayed after infection. Moreover, astrocytes and neurons regulate microglial function via release of various soluble signaling molecules suggesting the important role of cellular interactions (Borst et al., 2021). Despite the immune privilege of the CNS, pathogens can lead to autoimmune diseases through different mechanisms including e.g., the bystander activation of autoreactive cells or molecular mimicry. It has been proposed that initial antiviral responses are involved in the generation of myelin-specific CD4^+^ cells activated by epitopes that spread during the chronic phase, resulting in bystander myelin damage. In an experimental model of virus-induced demyelination, invasion of Theiler's murine encephalomyelitis virus (TMEV) into the CNS persistently infected the microglial cells and led to CD4^+^ T-cells-mediated immune response to myelin epitopes presented by microglia with the result of autoimmune demyelinating disease (Gerhauser et al., 2019). During demyelination, microglia can have both harmful and beneficial effects. In mice infected with a neurotropic coronavirus, microglia revealed a critical protective effect on the initiation of remyelination and the attenuation of immune-mediated demyelination (Sariol et al., 2020). Recent findings show a link between EBV and multiple sclerosis (MS) (Hassani et al., 2018; Bjornevik et al., 2022). EBV was detected as transcriptionally active in the white matter and meninges of the brain in most cases of MS (Hassani et al., 2018). In another study, EBV infection preceded MS onset and was associated with greatly increased disease risk, supporting the involvement of the virus in MS pathogenesis (Bjornevik et al., 2022). Astrocytes and microglial cells in MS patients were also infected by EBV (Hassani et al., 2018). The amoebic form was present in all microglia, indicating the activated state of these cells. All these findings suggest that microglia can regulate the virus-induced/associated demyelination through different mechanisms and that viral clearance in the CNS occurs most likely at the onset of neuroinflammation.

### 2.4 Protective and destructive effects of microglia

Both protective and destructive effects of microglia can be expected during the course of viral infections (Filgueira et al., 2021). Microglial response to inflammation with production of cytokines and chemokines is crucial for attracting peripheral immune cell populations such as monocytes, neutrophils, dendritic cells and T-cells into the CNS. Microglia and T-cells were present in the injured CNS in both the early and late post-injury phase, whereas other peripheral immune cells were detected in the CNS only in the early phase after nerve injury and subsequently disappeared (Jin and Yamashita, 2016). Reactivity of microglia surrounding infected neurons was associated besides the morphological changes with an increase of pro-inflammatory cytokines such as CXCL10, CCL5, IL-6 and TNF-α, which contribute to recruitment of T-cells (Jin and Yamashita, 2016). In lethal coronavirus encephalitis in mice, depletion of microglia using an inhibitor of colony-stimulating factor 1 receptor (CSF1R) resulted in exacerbation of infection and confirmed that microglia are involved in inhibition of virus replication during the early phase after infection, with an impact on subsequent survival and morbidity/mortality. Moreover, the T-cell recruitment was insufficient after microglia depletion, supporting the critical role of microglia in the early innate and virus-specific T-cell responses and in the host defense against viral encephalitis (Wheeler et al., 2018). Also in mice infected with neuroinvasive WNV, reactive microglia controlled viral growth and reduced mortality (Stonedahl et al., 2020). Microglia can directly identify WNV viral particles in the CNS through various signaling pathways such as TLR3, resulting in antiviral response with cytokine release and recruitment of CD8^+^ T-cells important for the limitation/termination of the infection. Expression of matrix metalloproteinases such as MMP9 and intercellular adhesion molecule ICAM-1 by microglia may contribute to WNV invasion into the CNS by disrupting the BBB and leakage of potentially infected leukocytes into the brain (Stonedahl et al., 2020). In the long term, the recruited antiviral CD8^+^ T-cells differentiated in memory CD8^+^ T-cells producing IFN-γ and sustained in the virus-cleared CNS (Garber et al., 2019). IFN-γ signaling was necessary for microglial reactivity, which contributed to synaptic damage/synaptic elimination with involvement of complement-dependent mechanisms, leading to neurological impairment during recovery from WNV encephalitis (Garber et al., 2019; Stonedahl et al., 2020). The WNV-induced memory deficits were effectively prevented by microglia-specific deletion of IFN-γ receptor, suggesting that the interaction between T-cells and microglia is a key event involved in the development of post-infectious cognitive dysfunction after recovery from Flavivirus encephalitis (Garber et al., 2019). In addition, type-I IFN receptor signaling of astrocytes and neurons is also involved in microglial reactivity during viral encephalitis (Chhatbar et al., 2018).

### 2.5 Role of microglia in COVID-19

Recently, brain neurotropism has been demonstrated for SARS-CoV-2, inducing the Coronavirus Disease 2019 (COVID-19) pandemic (Tremblay et al., 2020). Many cell types in the CNS, including endothelial cells, neurons and glial cells such as astrocytes and microglia, express receptor angiotensin-converting enzyme 2, to which the SARS-CoV-2 binds (Tremblay et al., 2020). Neuropathological findings of COVID-19 cases were related to dysfunctional interactions between microglia and T-cells, microglial nodules in the perivascular CNS compartment as well as significantly higher microglial reactivity in the brain stem (Theoharides and Kempuraj, 2023). Furthermore, microglia and astrocyte subpopulations during COVID-19 infection corresponded to pathological cell states that have been previously detected in human neurodegenerative disease (Yang et al., 2021). The additional involvement of microglia in neurodegenerative processes emphasizes the complex role of microglia in the continuum between neuroinflammation and neurodegeneration (González-Scarano and Baltuch, 1999; Hickman et al., 2018).

## 3 Conclusions

In summary, the existing evidence support our opinion that microglia are a key crucial factor in the development of viral-induced neuroinflammation, regulating the initiation of the innate and adaptive immune responses with an impact on viral replication, spreading and elimination. In line with this, depending on the type of microglial response, the course of viral-induced neuroinflammation could be defined e.g., direct infection with acute or sustained neuroinflammation or bystander activation, impact on demyelination etc. Future research should focus on microglial profiles associated with distinct disease courses and signaling pathways involved in intercellular interactions of microglia during viral CNS infection.

## Author contributions

FI: Conceptualization, Data curation, Formal analysis, Methodology, Project administration, Supervision, Writing – original draft, Writing – review & editing. TF: Data curation, Writing – original draft. PF: Writing – review & editing. FC: Data curation, Writing – original draft.
